# The relationship of myocardial oxygenation to coronary flow and oxygen saturation during CO_2 _manipulations

**DOI:** 10.1186/1532-429X-16-S1-O110

**Published:** 2014-01-16

**Authors:** Kady Fischer, Dominik P Guensch, Nancy Shie, Matthias G Friedrich

**Affiliations:** 1 Philippa & Marvin Carsley CMR Centre at the Montreal Heart Institute, Montreal, Quebec, Canada; 2 Department Anesthesiology and Pain Medicine, Inselspital Bern, University of Bern, Bern, Switzerland

## Background

Oxygenation-sensitive (OS) imaging relies on alterations of deoxyhemoglobin (dHb) in the blood. Thus signal intensity (SI) is dependent on the factors that shift the dHb fraction; oxygen saturation, tissue oxygen extraction and blood supply. We aimed to physically measure and compare these parameters while manipulating myocardial oxygenation with systemic CO_2 _changes. These changes cause hypercapnic vasodilation and hypocapnic vasoconstriction, which affect the myocardial blood supply and subsequently myocardial oxygenation.

## Methods

In 8 anaesthetized swine, we acquired OS images in 3 short axis slices (mid, mid-apical and apical) using an ECG triggered balanced SSFP sequence with standard cine imaging for function acquired in a short-axis stack in a clinical 3T magnet during normoxia (paO_2 _= 100 mmHg). Arterial pCO_2 _levels of 30, 40 and 50 mmHg were targeted through ventilation settings. Coronary flow was measured using a surgically implanted perivascular MRI-compatible ultrasound flow probe around the left anterior descending (LAD) coronary artery. Blood gases were analyzed from blood drawn through a catheter in the femoral artery and in the coronary sinus, which were obtained simultaneously with LAD flow measurements and OS images. The images were analyzed in systole and assessed for the global myocardial %-change SI in comparison to the baseline values at paCO_2 _= 40 mmHg.

## Results

**Figure 1:**
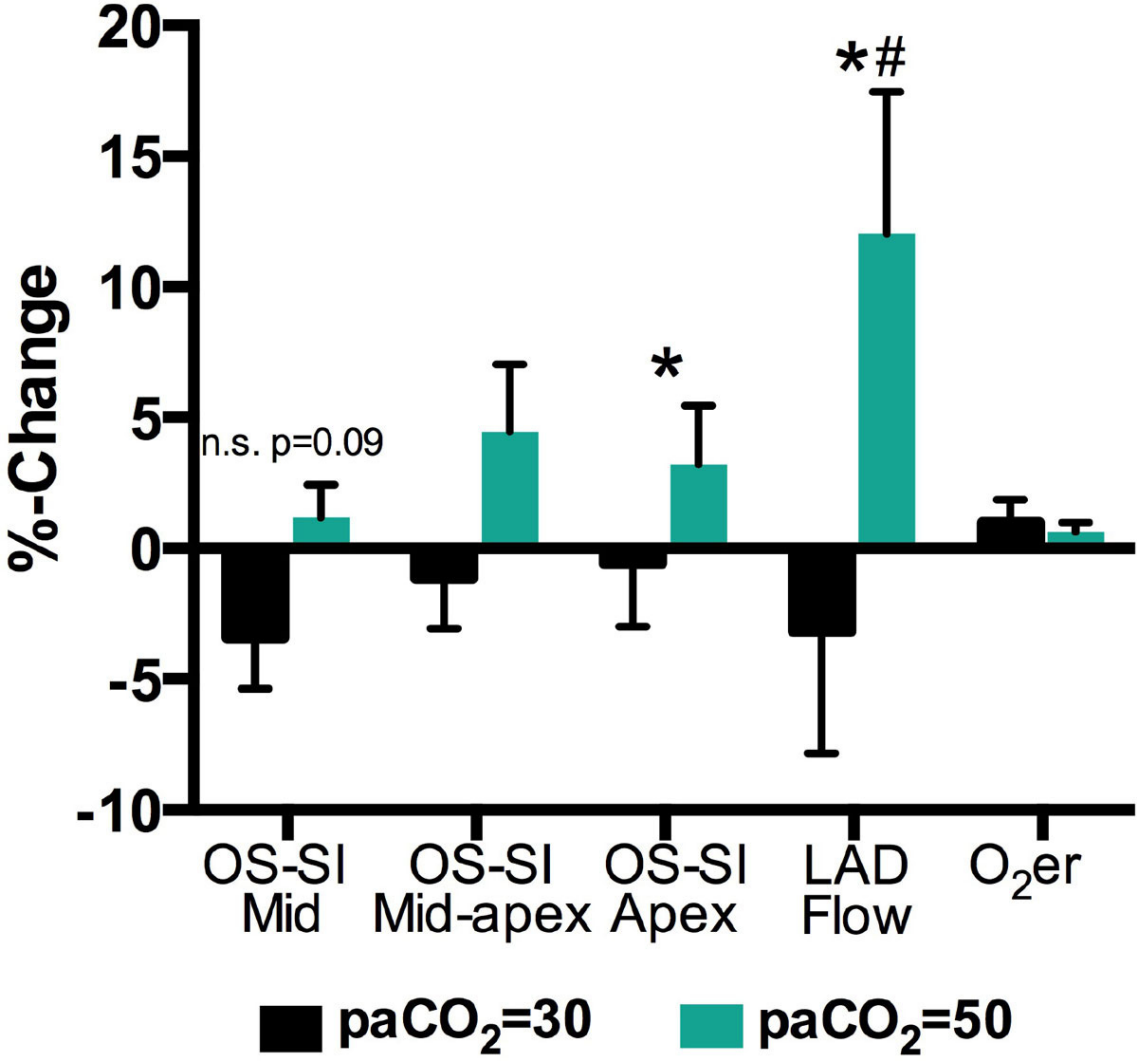
Mean ± SEM %-change of values from the baseline level at normoxia and normocapnia to levels with paCO_2 _= 30 mmHg (black) and paCO_2 _= 50 mmHg (green) (#p < 0.05 vs. baseline, *p < 0.05 difference between paCO_2 _of 30 and 50). Oxygenation-sensitive signal intensity (OS-SI) of the three SAX slices (n = 8), the blood flow in the LAD (n = 8), and the oxygen extraction ratio (O_2_er, n = 7).

OS-SI increased significantly along with LAD flow during hypercapnia (paCO_2 _= 50 mmHg) in comparison to hypocapnia (paCO_2 _= 30 mmHg, Figure 1). A decreasing trend was noted in both flow and SI for the hypocapnic levels in comparison to baseline (non-significant). Changes in oxygen extraction, saturation of both the arterial and coronary sinus blood, ejection fraction, heart rate and cardiac output were not different from baseline values.

## Conclusions

As oxygen extraction remained consistent during our maneuvers, the changes in OS-SI are explained by the increase in coronary blood flow, thus demonstrating the potential of CO_2_-induced vasomotor response in combination with oxygenation-sensitive imaging of the myocardium.

## Funding

Funding is provided by the Montreal Heart Institute Foundation and the Canadian Foundation for Innovation.

